# Re-Visiting Insecticide Resistance Status in *Anopheles gambiae* from Côte d'Ivoire: A Nation-Wide Informative Survey

**DOI:** 10.1371/journal.pone.0082387

**Published:** 2013-12-16

**Authors:** Alphonsine A. Koffi, Ludovic P. Ahoua Alou, Jean-Paul K. Kabran, Raphael N'Guessan, Cédric Pennetier

**Affiliations:** 1 Institut Pierre Richet (IPR), Bouaké, Côte d'Ivoire; 2 Laboratoire de Zoologie et Biologie Animale, Université Félix Houphouët Boigny, Abidjan, Côte d'Ivoire; 3 London School of Hygiene and Tropical Medicine, Keppel Street, London, United Kingdom; 4 Institut de Recherche pour le Développement (IRD), Maladies Infectieuses et Vecteurs, Ecologie, Génétique, Evolution et Contrôle (MIVEGEC), UM1-UM2-CNRS 5290 IRD 224, Montpellier, France; 5 Centre de Recherche Entomologique de Cotonou (CREC), Cotonou 04, Bénin; Université Pierre et Marie Curie, France

## Abstract

Insecticide resistance constitutes a major threat that may undermine current gain in malaria control in most endemic countries. National Malaria Control Programmes (NMCPs) need as much information as possible on the resistance status of malaria vectors and underlying mechanisms in order to implement the most relevant and efficient control strategy. Bioassays, biochemical and molecular analysis were performed on *An. gambiae* collected in six sentinel sites in Côte d'Ivoire. The sites were selected on the basis of their bioclimatic status and agricultural practices. *An. gambiae* populations across sites showed high levels of resistance to organochloride, pyrethroid and carbamate insecticides. The *kdr* and *ace-1^R^* mutations were detected in almost all sentinel sites with mosquitoes on the coastal and cotton growing areas mostly affected by these mutations. At almost all sites, the levels of detoxifying enzymes (mixed-function oxidases (MFOs), non-specific esterases (NSE) and glutathione-S-transferases (GSTs)) in *An. gambiae* populations were significantly higher than the levels found in the susceptible strain Kisumu. Pre-exposure of mosquitoes to PBO, an inhibitor of MFOs and NSEs, significantly increased mortality rates to pyrethroids and carbamates in mosquitoes but resistance in most cases was not fully synergised by PBO, inferring a residual role of additional mechanisms, including *kdr* and *ace-1* site insensitivity. The large distribution of resistance in Côte d'Ivoire raises an important question of whether to continue to deploy pyrethroid-based long-lasting insecticidal nets (LLINs) and insecticide residual spraying (IRS) towards which resistance continues to rise with no guarantee that the level of resistance would not compromise their efficacy. Innovative strategies that combine insecticide and synergists in LLINs or spatially LLIN and an effective non-pyrethroid insecticide for IRS could be in the short term the best practice for the NMCP to manage insecticide resistance in malaria vectors in Côte d'Ivoire and other endemic countries facing resistance.

## Background

Malaria vector control programmes rely largely on long-lasting insecticidal nets (LLINs) and indoor residual spraying (IRS) in sub-Saharan Africa [1] . For LLINs thirteen brands impregnated with only three pyrethroid insecticides (permethrin, deltamethrin and α-cypermethrin) are recommended by the World Health Organization Pesticide Scheme (WHOPES) [2]. WHOPES also recommends 12 insecticide formulations for IRS, involving four insecticide classes: organochlorides, organophosphates, carbamates and pyrethroids [3].

Unfortunately insecticide resistance mechanisms are spreading faster than thought across Africa [4]. This threatens to undermine the efficacy of IRS and LLINs that contain the four insecticide classes cited above. Four types of resistance mechanisms against public health insecticides have been identified: metabolic resistance, target site resistance, penetration resistance and behavioural resistance [4]–[6]. Nevertheless only metabolic and target site resistance mechanisms have been extensively investigated at both the phenotypic and genetic levels [7].

So far, all insecticides used in malaria vector control are neurotoxic. The acetylcholinesterase which hydrolyses the neurotransmitter, acetylcholine in the synaptic gap is the target of organophosphate and carbamate insecticides. The voltage-gated sodium channel that triggers impulse along the neuron membrane via discharge of Na^+^ is the target site of pyrethroid and organochlorine insecticides. The c-aminobutyric acid (GABA) is a neurotransmitter of the inhibitory synapses in the insect nervous system [8]. Non-silent point mutations that render these targets less sensitive to insecticides are respectively: the *ace-1^R^*, the *kdr* and the *Rdl* mutation [9]–[11]. Three main groups of enzymes are involved in metabolic resistance mainly through an overproduction process: carboxylesterases (efficient against organophosphate and carbamate insecticides), glutathione-S-transferases or GSTs (efficient against organophosphate, organochlorine, and pyrethroid insecticides) and cytochrome P450-dependent monooxygenases (efficient against most insecticide classes, frequently in conjunction with other enzymes). The overproduction might be the result of gene amplification increasing the gene's copy number or gene expression modification.

These mechanisms might have strong negative impact on the operational efficacy of both ITNs and IRS [12]–[14]. The study of forces driving these mechanisms evolution, their detection and their monitoring are crucial in order to implement the most relevant insecticide management strategy.

National Malaria Control Programmes (NMCP) in every endemic country have to revise and update their strategic plan every 3 or 5 years. In Côte d'Ivoire, the national strategy for malaria vector control relies essentially on the scaling up of systematic distribution of LLINs. Between 2007 and 2011 the NMCP distributed 9,671,246 LLINs to the targeted population of pregnant women and children under five years. Since 2011 the LLINs distribution campaign with 7,429,470 LLINs aims to scale the universal coverage up. The NMCP also plans to implement a vast IRS campaign to supplement universal coverage of LLINs [15].

In Cote d'Ivoire, there have been a number of studies investigating insecticide resistance mechanisms but they were all far from being representative because just two bioclimatic zones (guinean and wet savannah) were surveyed [16]–[20]. In order to implement the most relevant strategy in all bioclimatic zones, the NMCP developed and supported the present study to generate wider baseline data on resistance status in malaria vector populations in the country. The resistance levels to the four classes of insecticide so far deployed for ITNs and potentially for IRS in the country were investigated. The underlying mechanisms driving resistance were characterized.

## Methods

### Ethics statement

No specific permissions were required for the larvae sampling in urban areas because mosquito breeding sites were located in public areas. In contrast in rural areas, owners of the sampled cultivated areas gave their permission before the larvae collections. Field collections did not involve endangered or protected species.

### Study sites

**Figure 1:**
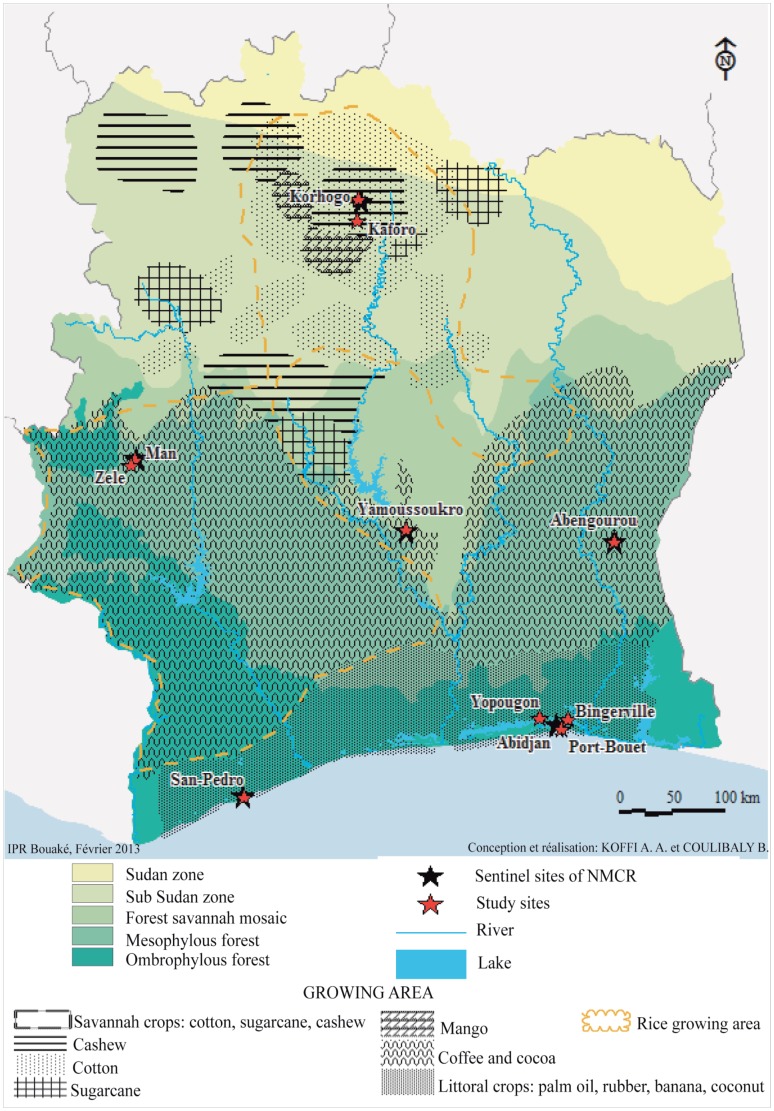
Map of Côte d'Ivoire showing the localities in the different ecological zones where anopheline mosquitoes were collected.

The study was conducted in six sentinel sites currently involved in malaria surveillance by the NMCP in Côte d'Ivoire. These sites were selected on the basis of bioclimatic parameters and agricultural practices in order to capture a broader range of ecological patterns found in Côte d'Ivoire (Figure 1 and Table 1). Where possible, mosquito larvae were sampled in breeding sites from both the urban and rural within sentinel sites:

**Table 1:** Description of sampling sites, agricultural practices, rural/urban status, bioclimatic zones and the distribution of the molecular forms of *An. gambiae s.s*.

Localities	Bio-climate status	Longitude	Latitude	Status	Main agricultural practices	Molecular forms		N
						S (%)	M (%)	
Korhogo	sub-sudannian	−5.626144	9.450654	urban	Rice, cotton, vegetables	23 (71.9)	9 (28.1)	32
Kaforo	sub-sudannian	−5.685582	9.277895	rural	Rice, cotton, vegetables	21 (65.6)	11 (34.4)	32
Yamoussoukro	pre-forest	−5.6627248	6.820336	urban	Rice, vegetables	5 (16.1)	26 (83.9)	31
Man	rain forest, montains	−7.562108	7.412094	urban	Cocoa, coffee, rice	14 (43.8)	18 (56.2)	32
Zele	rain forest, montains	−7.58967	7.36381	rural	Cocoa, coffee, rice	25 (78.1)	7 (21.9)	32
Abengourou	rain forest	−3.496321	6.729096	urban	Cocoa, coffee, rice	3 (9.4)	29 (90.6)	32
San-Pedro	rain forest	−6.616667	4.733333	urban	Rubber, palm trees, coconut palms, vegetable		31 (100)	31
Bingerville	rain forest	−3.90043	5.35929	urban	Vegetables	11 (34.4)	21 (65.6)	32
Port-Bouët	rain forest	−3.919482	5.24823	urban	Vegetable, horticultural		32 (100)	32
Yopougon	rain forest	−4.021887	5.324857	urban	Vegetables		32 (100)	32

San Pedro and Abidjan, belonging to the Guinean bioclimatic zone were characterized by hyper-ombrophilous rainy forest. The average annual rainfall is between 1400 mm and 2000 mm, with two rainy seasons in a year (April to July and September to November) with an average temperature of 26±2°C according to records from the local wheather stations. The main agricultural practices include coffee, cocoa, rubber, palm and coconuts. Breeding sites investigated at San Pedro and three localities selected within the economic capital city, Abidjan, (i.e. Yopougon, Port-Bouët and Bingerville) were situated in urban or peri-urban areas.
Man, is a city located in the Western zone of the country consisting of tropical rainy forest with hills and mountains. It is characterized by a single rainy season starting in February up to November. The average annual rainfall ranged from 1600 mm to 2500 mm and the annual temperature averaged 24.5°C. Coffee and cocoa are grown but at a small scale. There are valleys suitable for the production of rice. A nearby rural area, Zele, with intense farming was also considered for investigation.
Abengourou, situated in the Eastern part of Côte d'Ivoire has a Guinean bioclimatic profile too. It has one rainy season with an average annual rainfall between 1200 mm and 1700 mm and an annual average temperature of 25.8°C. Coffee and cocoa are the main cash crops produced in the area. The breeding sites surveyed in Abengourou are all from urban areas.
Yamoussoukro, located in the centre of the country is a transitional zone characterized by a wet savannah. This region has one rainy season and an average annual rainfall of 1200 mm and an annual average temperature of 25.8°C. Quantities of rice and vegetables are produced in the area for local consumption Breeding sites sampled there derived both from urban and sub-urban areas.
Korhogo, in the North is a city located in a sub-Sudanian bioclimatic zone. The rainy season generally starts between May-June, peaks in August and ends in late October. The mean annual rainfall generally plateaus at 1200 mm with temperature varying over the seasons and the bioclimatic zone matching the savannah type. Cotton is the major commercial cash crop grown in this area. Some vegetables, rice and mangoes are also grown but at a small scale. Kaforo a nearby rural area where cotton is also intensively produced was included.

### Mosquito collection

The sampling of *An. gambiae* larvae was done between June and July 2012 at the different sentinel sites (urban and rural). Larvae were collected from natural breeding sites such as ponds, puddles, footprints maintained by rainfall, and in rice and vegetable farms. They were then brought to the insectary at Institut Pierre Richet (IPR) and reared to adults.

### Insecticide susceptibility tests

Bioassays on adult mosquitoes were conducted using WHO test kits [21]. Filter papers were impregnated with diagnostic concentrations of eight insecticides from 4 distinct classes as follows:

Pyrethroids: permethrin (0.75%), deltamethrin (0.05%) and α-cypermethrin (0.05%);
Pseudo-pyrethroid: etofenprox (0.05%);
Organochlorides: Dichlorodiphenyltrichloroethane (DDT) (4%) and dieldrin (4%);
Carbamate: carbosulfan (0.4%);
Organophosphate: pyrimiphos-methyl (1%).

Bioassays were performed with batches of 25 unfed females of *An. gambiae*, 2–3 days old (four replicates per concentration). Mosquitoes were exposed to insecticide-impregnated papers for 60 min at 25±2°C and 80% relative humidity (RH). At the end of the exposure period, mosquitoes were transferred to observation tubes, supplied with 10% honey solution and held for 24 h before scoring delayed mortality. Batches of mosquitoes from each site exposed to untreated papers were used as negative controls.

In order to assess the involvement of detoxifying enzymes in the resistance phenotypes, complementary tests were performed with a 1 hour pre-exposure to piperonyl butoxide (PBO), an inhibitor of multiple function oxidases (MFO) and non-specific esterases (NSE). The wild mosquito populations were compared to the susceptible reference strain of *An. gambiae s.s.* Kisumu. All control survivor specimens (including the susceptible reference mosquito) were stored at −80°C for biochemical analysis. All samples exposed to the different insecticides were kept individually with silicagel at −20°C for molecular analysis.

### Identification of sibling species and M and S molecular of *An. gambiae s.s.*

Ribosomal DNA was extracted from individual mosquitoes following Collins *et al* [22] and used for polymerase chain reaction (PCR) analysis to determine the species from the *An. gambiae* complex following Scott *et al* [23] and M or S molecular form of *An. gambiae s.s.* according to Favia*et al* [24].

### PCR detection of the L1014F and L1014S *kdr* and G119S *ace-1* mutations

The presence of L1014F and L1014S *kdr* alleles was assessed using hot oligonucleotide ligation assay (HOLA) technique according to the protocol of Lynd *et al* [25]. The PCR-RFLP diagnostic test was used to detect the presence of G119S mutation (*ace-1* gene) as described by Weill *et al* [26].

### Biochemical analysis

Biochemical assays were performed to compare the amount of MFO and the activity levels of NSE for both ß and α-naphtyl acetate. The production of glutathione S-transferases (GST) was also investigated in all field samples relative to the susceptible Kisumu strain [27]. Mosquitoes used for the biochemical analysis have not been exposed to any insecticides prior to the assay.

### Data analysis

WHO criteria [28] were adopted to define the resistance status of the mosquito populations. When less than 80% mortality was observed the population was considered ‘resistant’, between 80% and 97% mortality the population was considered ‘tolerant’ (or ‘suspected of resistance’) and when the mortality was above 98% the population was considered ‘susceptible’. Biochemical assay data (amounts of MFO and enzymatic activity per mg protein of NSE and GST) of Kisumu and the *An. gambiae* populations from the sentinel sites were compared using Kruskall-Wallis non-parametric test (Statistica software). Conformity of L1014F and L1014S *kdr* and *ace-1* mutations frequencies with Hardy-Weinberg equilibrium was tested for *An. gambiae* population from the sentinel sites using the exact probability test [29]. The frequency of the duplicated allele *ace-1^D^* was estimated as described in Lenormand *et al* [30]. Statistical significance was set at the 5% level.

## Results

### Species and molecular forms of *An. gambiae* in Côte d'Ivoire

Table 1 illustrates the distribution of M and S *An. gambiae s.s.* molecular forms at the different sentinel sites. In 318 mosquito samples analysed for sibling species identification across the eco-geographical sentinel sites, only *An. gambiae s.s.* were found. The S molecular form was predominant in the sub-Sudanian area in the north both in urban and rural areas (71.9% in Korhogo and 65.6% in Kaforo) and in the rural area of Zele (78.1%) in the Western part of Côte d'Ivoire. The distribution in the neighbouring urban area of Man was different, the S form representing 43.8% only of the *An. gambiae* population. By contrast, in the pre-forested (Yamoussoukro) and the rain forest areas (San Pedro, Bingerville, Yopougon, Port-Bouët), the M form was predominant (65.6% in Bingerville and 100% in San-Pedro, Yopougon and Port-Bouët).

### Resistance status

**Figure 2:**
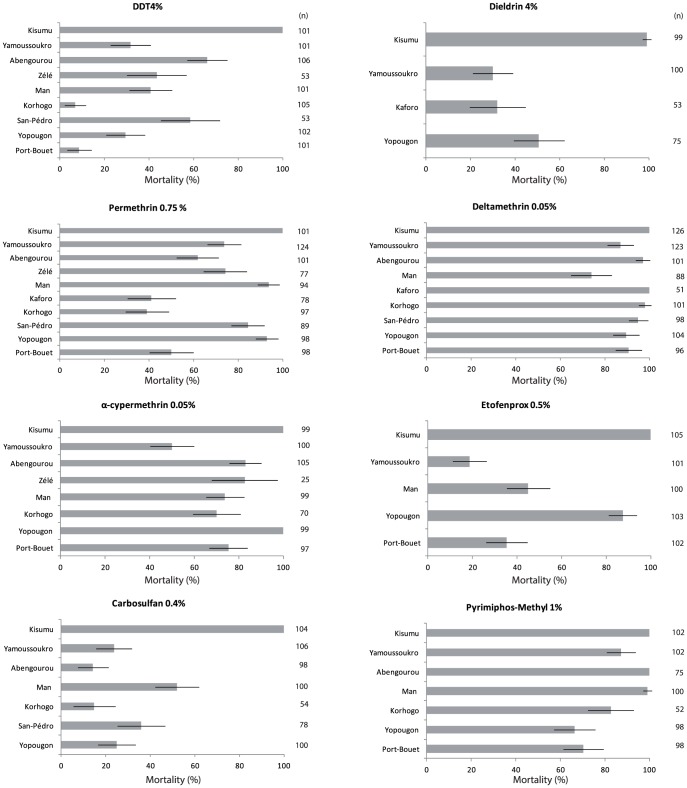
Insecticidal effects of diagnostic concentrations of insecticides against Anopheles gambiae mosquitoes from different sentinel sites (60 min contact in WHO tube tests).

Figure 2 shows the insecticide resistance status of *An. gambiae* populations from the sentinel sites compared with the susceptible reference strain Kisumu. All insecticides tested killed 99–100% of susceptible mosquitoes indicating the accuracy of the active ingredient deposits on the filter papers used for the bioassays.

*An. gambiae* populations from all sites showed strong resistance to permethrin and DDT with some levels of tolerance to permethrin observed in the urban areas of Man, San-Pedro and Yopougon. Dieldrin resistance was reported in the few representative sites tested (Man, Yopougon, Yamoussoukro and Kaforo) (Fig. 2). Populations from all 10 sites were more vulnerable to deltamethrin than any other insecticides tested; mortality ranged from 74–100% between locations, with Korhogo and Kaforo (cotton areas) showing susceptibility (98–100% mortality) despite they displayed strong resistance to permethrin and DDT.

There was resistance to alpha-cypermethrin at all sites, except populations from Yopougon. A high frequency of resistance to etofenprox was recorded in all populations of *An. gambiae s.s.* (Fig. 2).

*An. gambiae s.s.* was strongly resistant to carbosulfan (14–52% mortality) at all site but showed reduced susceptibility to pyrimiphos-methyl in Yamoussoukro, Korhogo, Yopougon and Port-Bouët (66–87%) and full susceptibility in Abengourou and Man (99–100%).

### Detection of resistance genes by PCR

The frequency of the *kdr* mutations was investigated in every sample from all sentinel sites. The distribution of the L1014F *kdr* mutation in the molecular forms of *An. gambiae s.s.* populations is shown in Table 2. The L1014F *kdr* was detected in both M and S molecular forms. Its average frequency ranged from 0.47 to 0.86 with the highest values (>0.70) reported in the coastal localities (Bingerville, Port-Bouët, San-Pedro) and the areas with cotton in the North (Korhogo, Kaforo). No L1014S *kdr* type was detected in all samples analysed (N = 317).

**Table 2:** Distributionof genotypes and allelic frequency of the *L1014F kdr* mutation in *An.gambiaes.s.*populations from sentinel sites.

	Molecular form											
	M					S						
	n1	SS	RS	RR	f(R)	n2	SS	RS	RR	f(R)	n1+n2	f(R)
Bingerville	21	1	3	17	0.881	11	2	3	6	0.682	32	0.813
Port-Bouët	31	3	3	25	0.855						31	0.855
Yopougon	31	2	29	0	0.468						31	0.468
Yamoussoukro	26	4	17	3	0.479	5	0	5	0	0.500	31	0.484
Korhogo	9	0	5	4	0.722	23	0	11	12	0.761	32	0.750
Kaforo	10	0	4	6	0.800	20	0	7	13	0.825	30	0.817
Man	15	2	13	0	0.433	11	0	10	1	0.545	26	0.481
Zele	7	0	7	0	0.500	23	0	23	0	0.500	30	0.500
San-Pedro	32	4	10	18	0.719						32	0.719
Abengourou	29	3	22	4	0.517	3	0	2	1	0.666	32	0.531

n: number analysed; f(R): frequency of the mutation.

The G119S mutation conferring resistance to organophosphates and carbamates was detected in 4 localities out of 6 in both molecular forms (Table 3). The M and S specimens carrying the mutation were heterozygotes, except for 5 specimens out of 26 (19.2%) found homozygous in Korhogo in the North and 1 out of 12 (8.3%°) in Man in Western Côte d'Ivoire. Based on the Hardy-Weinberg equilibrium, this excess of heterozygous supports the presence of a duplicated allele (*ace-1^D^*) in *An. gambiae s.s.* from the sentinel sites. Table 3 shows the predicted distribution of the *ace-1^D^* in the S and M forms of *An. gambiae s.s.*. The presence of the *ace-1^D^* is suspected in either M or S forms of *An. gambiae s.s.* from all sites except Abengourou and San-Pedro (Table 3). It is only significantly supported (after Bonferroni correction) in M samples from Port-Bouët and S from Bingerville, the model suggesting a frequency around 0.47 for those from Port-Bouët (p<0.01) and 0.57 from Bingerville (p = 0.01).

**Table 3:** *ace-1* allele frequency distribution in *An. gambiae s.s.* molecular forms from Côte d'Ivoire.

	M molecular form					S molecular form				
Localities	N	S	R	D	F_HW_	N	S	R	D	F_HW_
Bingerville	20	0.74	0.00 [0-0.301]	0.26 [0-0.420]	0.10	11	0.43	0.00 [0-0.400]	**0.57 [0.182-0.819]**	0.01*
Port-Bouët	32	0.53	0.00 [0-0.241]	**0.47 [0.251-0.617]**	0.00*	0				
Yopougon	32	0.81	0.00 [0-0.235]	0.19 [0-0.304]	0.13	0				
Yamoussoukro	26	0.85	0.00 [0-0.225]	0.15 [0-0.262]	0.30	5	0.89	0.00 [0-0.367]	0.11 [0-0.390]	0.74
Korhogo	8	0.94	0.00 [0-0.245]	0.06 [0-0.255]	0.80	23	0.72	0.28 [0.122-0.422]	0.00 [0-0.228]	1.00
Kaforo	9	0.61	0.39 [0.180-0.617]	0.00 [0-0.299]	1.00	21	0.76	0.22 [0.053-0.379]	0.03 [0-0.264]	0.82
Man	17	0.87	0.00 [0-0.241]	0.13 [0-0.269]	0.46	14	0.85	0.00 [0-0.284]	0.15 [0-0.325]	0.41
Zele	7	1.00	0.00 [0-0.128]	0.00 [0-0.128]	1.00	25	0.72	0.20 [0.048-0.381]	0.08 [0-0.316]	0.46
San-Pedro	32	1.00	0.00 [0-0.030]	0.00 [0-0.030]	1.00	0				
Abengourou	29	1.00	0.00 [0-0.033]	0.00 [0-0.033]	1.00	3	1.00	0.00 [0-0.274]	0.00 [0-0.274]	1.00

N: number analysed; Values in brackets represent the confidence interval at 95%

S, R and D represent the estimated frequencies of the susceptible, resistant and duplicated alleles respectively. The probability of the departure from Hardy-Weinberg expectation (*F*_HW_) is bold when significant and has a star when still significant after Bonferroni correction.

### Synergist and biochemical analysis

#### Synergist

**Figure 3:**
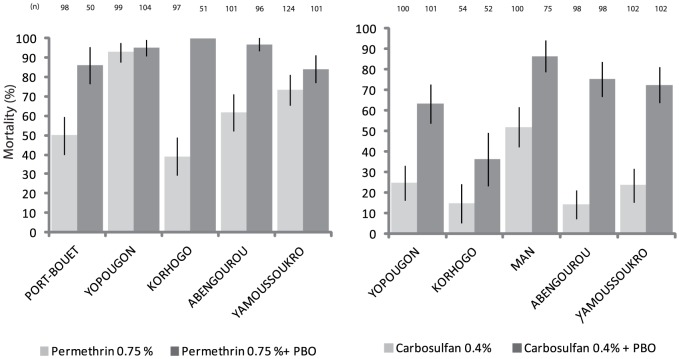
Insecticidal effects of diagnostic concentrations of one pyrethroid insecticide (permethrin) and one carbamate insecticide (carbosulfan) (60 min contact in WHO tube tests) with or without a 60 min pre-exposition to PBO.

Figure 3 shows the toxicity of permethrin and carbosulfan with and without PBO against *An. gambiae s.s.* from the sentinel sites. Pre-exposure of mosquitoes to PBO significantly increased the mortality rates to permethrin from all locations (p<0.05) except Yopougon (p = 0.539) (Figure 3a). Interestingly, PBO increased mortality to permethrin to full susceptibility level in populations from Korhogo (from 39% to 100%) and Abengourou (62% to 97%), suggesting that the resistance phenotype in these two areas is almost entirely mediated by the metabolic activities of MFOs and NSE.

Resistance to carbosulfan was largely pronounced across sites, mortality ranging from 14% to 63%. Pre-exposure of mosquitoes to PBO significantly increased mortality rates to carbosulfan at all sites but resistance was not fully synergised by this MFO and NSE inhibitor, inferring a residual role of additional mechanisms, including the *ace-1* site insensitivity.

#### Biochemical assays

Table 4 compares the mean amount of MFO and the mean activities of the NSE and GST found in *An. gambiae s.s.* from the sentinel sites relative to the levels in the susceptible *An. gambiae* Kisumu.

**Table 4:** Mean level of NSE. MFO and GST activity in *An. gambiae s.s.* populations from the sentinel sites relative to the susceptible reference strain Kisumu.

	NSE						MFO			GST		
Localities	N	μmol α-naphthol/min/mg protein	AR	N	μmol ß-naphthol/min/mg protein	AR	N	nmol P450/mg protein	QR	N	nmol GSH conj/min/mg protein	AR
Kisumu	40	0.086±0.007		40	0.084±0.007		38	0.095±0.008		40	0.295±0.032	
Yopougon	32	**0.352**±**0.054**	**4.1**	32	**0.313**±**0.058**	**3.7**	32	**0.255**±**0.048**	**2.7**	24	**2.358**±**0.679**	**8**
Port-Bouët	17	**0.300**±**0.070**	**3.5**	15	**0.365**±**0.171**	**4.2**	12	**0.236**±**0.044**	**2.5**	14	**1.065**±**0.308**	**3.6**
Korhogo	32	**0.340**±**0.052**	**4.0**	32	**0.206**±**0.031**	**2.5**	23	**0.209**±**0.059**	**2.2**	21	**1.236**±**0.354**	**4.2**
Kaforo	15	**0.155**±**0.036**	**1.8**	15	0.105±0.027	**1.2**	15	**0.330**±**0.052**	**3.5**		-	
Yamoussoukro	33	**0.192**±**0.036**	**2.2**	33	0.138±0.028	**1.6**	32	**0.198**±**0.050**	**2.1**	32	**1.008**±**0.198**	**3.4**
Abengourou	32	0.036±0.011	**0.4**	32	0.043±0.009	**0.5**	31	**0.251**±**0.075**	**2.6**	25	0.501±0.268	**1.7**
Man	32	**0.150**±**0.024**	**1.7**	32	**0.175**±**0.050**	**2.1**	31	**0.420**±**0.067**	**4.4**	26	**0.664**±**0.142**	**2.3**
Zele	32	0.107±0.016	**1.2**	32	0.089±0.014	**1.1**	33	0.163±0.037	**1.7**	28	0.638±0.192	**2.2**
San-Pedro	40	**0.152**±**0.011**	**1.8**	40	**0.178**±**0.024**	**2.1**	39	**0.216**±**0.028**	**2.3**	34	0.471±0.084	**1.6**

N: total tested. Number in bold indicated samples where enzyme level or activity was significantly higher compared with Kisumu. (P<0.05) at the 5% level. AR: Activity Ratios. QR: Quantity ratios. GSH: Reduced form of glutathione.

All wild samples analyzed displayed significantly higher levels of esterase activity (using α-naphtyl acetate as a substrate) than that measured for Kisumu (p<0.05), except samples from Abengourou and Zele. The trend in activity for assays using ß-naphtyl acetate as a substrate was similar to the α-naphtyl assays. The highest α-and ß-esterase activities (>2.5-fold) were recorded on the coast (Yopougon, Port-Bouët) and the cotton growing area of Korhogo.

The production of MFO (cytochrome P450s) amount in *An. gambiae* from all sites but not Zele was significantly higher than the content measured in Kisumu (p<0.05), with broadly similar mean levels of activity expressed between locations.

The GST levels of activity in Yopougon, Port-Bouët, Korhogo, Yamoussoukro and Man populations were significantly higher than in Kisumu and the other field populations (p<0.05).

Overall a patchy distribution of overproduced quantities of NSE, MFO and GST was found in most *An. gambiae s.s.* populations analysed relative to the normal strain Kisumu; the most affected areas being on the coastal urban part of the country (Yopougon and Port-Bouët with vegetables and horticulture) and Korhogo in the North where cotton is produced.

## Discussion

A nation-wide survey of insecticide resistance in *An. gambiae s.s*. in Côte d'Ivoire was conducted in order to have an overview of the resistance status and establish a baseline dataset that would guide the National Malaria Control Programme.

The bioassays data showed high resistance levels of *An. gambiae s.s.* to organochlorides, carbamates and pyrethroids but at a lesser extent toward deltamethrin. The resistance levels to the organophosphate (pyrimiphos-methyl) varied greatly from susceptibility to resistance across sites. The *kdr* and *ace-1^R^* mutations were highly expressed in all *An. gambiae s.s.* populations with the exception for the *ace-1^R^* in samples from Abengourou and San-Pedro. The *ace-1^D^* duplication was present in Bingerville and Port-Bouët respectively in S and M molecular forms confirming previous finding highlighting its presence in Côte d'Ivoire [31], [32]. Its spread seemed to be wider as it was suspected in the urban areas of Yopougon, Yamoussoukro and Man although the low sample size did not allow to confirm it.

The MFO quantities and, NSE and GST activities in almost all *An. gambiae* populations were significantly higher than in the susceptible strain Kisumu and the bioassays data with PBO indicated that at least the MFO and NSE were involved in the phenotypic expression of the resistance.

This study provides a wider view of the spread of insecticide resistance in Côte d'Ivoire and adds a significant baseline knowledge to recent reports of high insecticide resistance levels in M'Bé [19], Yaokoffikro [20] near Bouaké in central Côte d'Ivoire, Tiassalé [18], [33] and Adzopé [17] in southern Côte d'Ivoire. All these reports including the present highlight the level of spread of resistance to all class of insecticides deployed for vector control, either as LLIN already in place or IRS under consideration for implementation in Côte d'Ivoire; an increasing trend commonly shared by several countries in West Africa[34]–[39].

The association between agricultural practices and the build up of insecticide resistance has been intensively investigated. It is worth noticing that *An. gambiae s.s.* population from areas with massive cotton production (Korhogo, Kaforo) displayed the highest resistance levels against almost all insecticides classes (organochlorides, pyrethroids and carbamates) as previously reported [34], [40]–[44]. Urban vegetable farming in Yopougon, Port-Bouët and Yamoussoukro areas was also associated with high levels of resistance, as previously detected [45]–[48]. The trend was not so clear within the rice-growing areas, generally associated with low application of insecticide [40], [44] but where moderate to high resistance level was found in *An.gambiae* [49].

There is a growing expansion of rubber production in Côte d'Ivoire and generally across part of Africa. So far reports on insecticide resistance in such context are scarce [50]. In-depth studies documenting the use of insecticides both socially and in the o agricultural sector is highly stressed in order to better appraise factors selecting and driving the evolution of insecticide resistance mechanisms [32].

The significance of the present study for the NMCP in Côte d'Ivoire raises an important question of whether to continue to deploy pyrethroid based LLINs and IRS towards which resistance continues to rise with no guarantee that the level of resistance seen in the country would not compromise their efficacy. No doubt it would be difficult to demonstrate the impact of resistance on the effectiveness of any of these interventions. There have been extensive randomized controlled trials (RCTs) (phase III) in part of Africa aiming at investigating the efficacy of ITNs for malaria prevention [14], [51] but very few have assessed how pyrethroid resistance might affect the effectiveness of such intervention. RCTs entail a set of communities randomly divided into groups, one that receives the novel form of vector control intervention, and comparison arms that often receive the old form of vector control tools or nothing. The key difficulty is that it is impossible to address the question to whether vector control would produce a smaller reduction in malaria if the vector mosquitoes are resistant than it would have done if they were susceptible, using RCT methods. This is simply because resistance is not an easy factor that can be allocated randomly to some communities and not to others. The distribution of resistance is patchy and its severity seems to differ from one area (locality) to another as seen in the present study. Moreover there may be more resistance or survival trend of mosquitoes in some villages than others because of variations in the quality of vector control operations [52] or in mosquito behavior [53]. This means there is no straight forward solution to address resistance impact. Some researchers are currently striving to bring up to the market novel insecticides or combination of these with pyrethroids to circumvent insecticide resistance but this may take several years before they are approved and made available for use. In the short term, NMCPs could consider adopting intervention strategies that e.g. combine a synergist such as PBO with pyrethroids in LLINs. The technology is intended to alleviate the load of resistance by removing the metabolic component of resistance due to MFOs and NSE [54]. The well known commercialized prototypes PermaNet® 3.0 [55]–[58] and more recently Olyset Plus® [59] of this kind have proved to be highly active against pyrethroid resistant mosquitoes and could be the product of choice for NMCPs. Ultimately, where LLINs are already in place and vectors survive them because of pyrethroid resistance [12], [13], NMCPs could consider spraying the homes with a non pyrethroid insecticide. In any event, insecticide combinations within homes for malaria control is an unavoidable reality. LLIN coverage is going universal and IRS with non-pyrethroid insecticides is being applied concurrently with LLINs as malaria control policy in many areas of high malaria transmission. Given the high resistance to carbamates detected across all sites but an appreciable level of susceptibility to the organophosphate, pyrimiphos methyl, it would be advisable to deploy this insecticide for IRS in combination with LLINs in Côte d'Ivoire. Recent evaluation of this insecticide applied as IRS in experimental huts showed extremely high level of control of pyrethroid resistant mosquitoes [60].

## Conclusion

The insecticide resistance data presented in the sentinel survey are of great interest for the NMCP of Côte d'Ivoire and beyond, to countries also facing similar rise in the level of pyrethroid resistance in their local vectors. Innovative strategies that combine insecticide and synergists in LLINs or spatially LLIN and effective insecticide for IRS within homes could be in the short term the best practice for NMCPs to manage insecticide resistance in malaria vectors in endemic countries.
